# Mycoprotein Production by Submerged Fermentation of the Edible Mushroom *Pleurotus ostreatus* in a Batch Stirred Tank Bioreactor Using Agro-Industrial Hydrolysate

**DOI:** 10.3390/foods12122295

**Published:** 2023-06-07

**Authors:** Georgios Bakratsas, Angeliki Polydera, Oskar Nilson, Alexandra V. Chatzikonstantinou, Charilaos Xiros, Petros Katapodis, Haralambos Stamatis

**Affiliations:** 1 Biotechnology Laboratory, Department of Biological Applications and Technologies, University of Ioannina, 45110 Ioannina, Greece; georgiosbakratsas@gmail.com (G.B.); apolyder@uoi.gr (A.P.); alexandra_xatzi@hotmail.com (A.V.C.); hstamati@uoi.gr (H.S.); 2 RISE Processum AB, SE-89122 Örnsköldsvik, Sweden; oskar.nilsson@metsagroup.com (O.N.); charilaos.xiros@processum.se (C.X.)

**Keywords:** single-cell protein, submerged cultivation, agro-industrial hydrolysate, amino acid metabolism, bioreactors

## Abstract

The demand for cheap, healthy, and sustainable alternative protein sources has turned research interest into microbial proteins. Mycoproteins prevail due to their quite balanced amino acid profile, low carbon footprint and high sustainability potential. The goal of this research was to investigate the capability of *Pleurotus ostreatus* to metabolize the main sugars of agro-industrial side streams, such as aspen wood chips hydrolysate, to produce high-value protein with low cost. Our results indicate that *P. ostreatus* LGAM 1123 could be cultivated both in a C-6 (glucose)- and C-5(xylose)-sugar-containing medium for mycoprotein production. A mixture of glucose and xylose was found to be ideal for biomass production with high protein content and rich amino acid profile. *P. ostreatus* LGAM 1123 cultivation in a 4 L stirred-tank bioreactor using aspen hydrolysate was achieved with 25.0 ± 3.4 g L^−1^ biomass production, 1.8 ± 0.4 d^−1^ specific growth rate and a protein yield of 54.5 ± 0.5% (g/100 g sugars). PCA analysis of the amino acids revealed a strong correlation between the amino acid composition of the protein produced and the ratios of glucose and xylose in the culture medium. The production of high-nutrient mycoprotein by submerged fermentation of the edible fungus *P. ostreatus* using agro-industrial hydrolysates is a promising bioprocess in the food and feed industry.

## 1. Introduction

Mycoprotein is a whole protein derived from filamentous fungi biomass. Because of its high nutritional value, mycoprotein can be used for meat analogues production. Apart from plant sources or GMO, meat analogues could also be produced from single-cell proteins derived from biomass of cultivated fungi or bacteria [1]. Fungal-derived proteins are gaining acceptance due to their low carbon footprint and high sustainability potential compared to conventional protein sources [2]. Lately, the upscaling efforts by various companies worldwide towards mycoprotein production have been multiplied, reflecting the demand of consumers for cheap, healthy, and sustainable alternative protein sources. Their quite balanced amino acid profile, with all the essential amino acids present, make mycoproteins suitable for both food and feed applications [3]. Besides their high quality, the cell walls of mycoproteins contain a “fibrous chitin–glucan matrix” (with a fiber content of at least 6%) making them a food source that is high in fiber. A review on human trials conducted to study the effect of consuming mycoproteins has shown that it could lead to a reduction of total cholesterol levels. Moreover, essential amino acids that increase muscle protein fractional synthesis levels have been reported for different mycoproteins [4]. Mycoprotein could also be produced by submerged cultivation of macrofungi [5,6].

Submerged cultivation of microorganisms is their cultivation in a liquid media containing carbon sources, nitrogen sources and micronutrients [7]. Because of the advantages of easy and rapid control of cultivation conditions, submerged cultivation prevails over fruiting bodies cultivation method in the industry as a way to produce bioactive mushroom compounds [8,9]. In addition, stirred-tank and air-lift bioreactors could also be used in submerged cultivation, making industrial applications more possible [7]. A wide variety of strains of known edible mushrooms could be cultivated in submerged conditions, and a wide range of bioactive compounds could be extracted from mushroom mycelia [10]. *Agrocybe aegerita*, *Pleurotus sapidus*, *Lentinula edodes*, *Stropharia rugosoannulata*, *Pleurotus sajor-caju* and *Pleurotus salmoneostramineus* have already been cultivated in liquid media for production of vegan protein sources [5]. Moreover, an *Agaricus bisporus* meat analogue revealed superior textural properties and umami characteristics compared to those of soy protein [6]. The optimization of single-cell production by submerged cultivation of *P. ostreatus* LGAM 1123 in a glucose-based medium has been studied [11].

To date, commercial mycoprotein production is based on simple sugars as the feedstock. A substrate ideal for mycoprotein production should contain enough nitrogen, carbon, and micronutrients for rapid mycelium growth [12]. Pea-processing byproduct, different pomaces (apple, pomegranate and aronia), leaf spinach and beet molasses have also been used for mycoprotein production [5,13]; however, sustainability and economic concerns have boosted the efforts to use lignocellulosic residual forms of plant biomass as feedstock. Such sources include agro-industrial residues, municipal solid waste, and several side streams from forestry industries, such as pulp and paper. These sources have the potential to lower both the production cost and the carbon footprint of mycoprotein [14]. The use of such raw materials demands their conversion to sugars prior to mycoprotein production, usually by enzymatic hydrolysis and the removal of the remaining solids prior to the cultivation [14,15,16]. Glucose is the most abundant sugar (60–70%) in lignocellulosic hydrolysates, whereas xylose follows with a value of 40%. Consequently, the simultaneous use of glucose and xylose by a microorganism increases the content of accessible sugars in lignocellulosic biomass to up to a value of 60 to over 90% depending on the type of the substrate [17]. Sugars contained in lignocellulosic biomass can be available after chemical and/or enzymatic hydrolytic treatment processes. More specifically, enzymatic hydrolysis led to a 72% saccharification yield for glucose and xylose [18]. Fungi that can metabolize both glucose and xylose have already been reported [19,20]. Proteomic analysis for *P. ostreatus* submerged cultivated in lignocellulosic polysaccharides, composed of pentoses, such as xylose, and hexoses, such as glucose, has already been reported [21]. Single-cell production by submerged cultivation of *P. ostreatus* LGAM 1123 in a glucose-based industrial side stream has also been reported by our group [11]. In addition, aspen biomass has gained attention due to the high content of holocellulose (80%), consisting of cellulose (50%) and hemicellulose (30%), and the low lignin content (20%), making it a valuable substrate for submerged cultivation of *Armillaria Gemina* [22]. To our knowledge, the combined use of glucose and xylose, which are the main sugars contained in agro-industrial side streams as substrates in submerged cultivation of fungi for the production of mycoproteins has not yet been studied.

In this work, *P. ostreatus* LGAM 1123 has been cultivated in a liquid medium for protein production using glucose and xylose monosaccharides separately or in combinations and in an agro-industrial hydrolysate from aspen wood chips. At first, different xylose concentrations were supplied in the growth medium to see how well the strain metabolizes pentose sugars. Then, to simulate different glucose/xylose ratios that could be found in natural substrates or agro-industrial side streams, different glucose and xylose ratios were added to the cultivation medium, to improve biomass and protein yield and estimate consumption rates of glucose and xylose. The protein quality as well as the potential differences in amino acid synthesis depending on different glucose/xylose ratios were investigated using amino acid analysis. Finally, we investigated the potential of an industrial application of the process with a reduced total cost. Mycoprotein was produced in a 4 L stirred tank bioreactor by batch cultivation of *P. ostreatus* LGAM 1123 in a cellulosic aspen wood hydrolysate obtained after enzymatic hydrolysis.

## 2. Materials and Methods

### 2.1. Chemicals and Reagents

Analytical grade chemicals were used in this study. Yeast extract and potato dextrose agar (PDA) were supplied from Neogen Europe Ltd. (Ayr, UK). Zinc sulphate heptahydrate (ZnSO_4_·7H_2_O), glucose, manganese (II) sulfate heptahydrate (MnSO_4_·7H_2_O), thiamine hydrochloride (Vitamin B1), ethylenediaminetetraacetic acid disodium salt dihydrate (EDTA-Na_2_·2H_2_O) (Sodium EDTA), fructose, xylose, maltose, peptone, bovine serum albumin (BSA), 4-(Dimethylamino) azobenzene-4′-sulfonyl chloride, (Dabsyl chloride), and phenol were supplied from Sigma-Aldrich (St. Louis, MO, USA). Sodium nitrate (NaNO_3_), di-Potassium hydrogen phosphate anhydrous (dibasic) (K_2_HPO_4_), potassium chloride (KCl), sodium nitrate (NaNO_3_), potassium nitrate (KNO_3_), ammonium chloride (NH_4_Cl) and ammonium sulfate ((NH_4_)_2_SO_4_) were supplied from AppliChem (Darmstadt, Germany). Magnesium sulfate anhydrous (MgSO_4_), calcium chloride dihydrate (CaCl_2_·2H_2_O), ferrous sulfate heptahydrate (FeSO_4_·7H_2_O), ammonium molybdate tetrahydrate ((NH_4_)Mo_7_O_2_·4H_2_O), sucrose, and urea were supplied from Fluka (Buchs, Switzerland). Sodium hydroxide (NaOH) was purchased from Panreac (Barcelona, Spain). Chloroform, acetonitrile and methanol and were supplied from Thermo Fisher Scientific (Waltham, MA, USA). Sulfuric acid was supplied from Honeywell Riedel-de Haën, and hydrochloric Acid (HCl) was supplied from Merck (KGaA Darmstadt, Darmstadt, Germany).

### 2.2. Microorganism

Ιn this study, the Laboratory of General and Agricultural Microbiology (Agricultural University of Athens, Athens, Greece) kindly provided *P. ostreatus* LGAM 1123 that was used for all the experiments. The strain was kept at 4 °C on potato dextrose agar (PDA) Petri dishes.

### 2.3. Media and Growth Conditions

For submerged precultures, 1 cm of the PDA Petri dishes cultures was transferred into a 250 mL Erlenmeyer flask using a sterilized cutter. The flasks contained 100 mL of the basal medium (g L^−1^), which consists of the following: glucose, 30, yeast extract, 10, NaNO_3_, 0.4, MgSO_4_, 1.15, K_2_HPO_4_, 0.7, KCL, 0.75, ZnSO_4_·7H_2_O, 0.0114, CaCL_2_·2H_2_O, 0.52, MnSO_4_·H_2_O, 0.03, FeSO_4_·7H_2_O, 0.03, (NH_4_)Mo_7_O_2_·4H_2_O, 0.01, Vitamin B1, 0.015, and Sodium EDTA, 0.75. The precultures incubated in a rotary shaker at 150 rpm and 28 °C, for twelve days. After that, 5 mL of culture was transferred into a new flask containing a slightly, depending on the experiment, modified basal medium. The initial pH of the medium was adjusted to 5.0 with the addition of 1 M NaOH prior to heat sterilization at 121 °C for 20 min. At different time, samples were withdrawn from the culture and centrifugated at 4000 rpm for 5 min to separate the biomass from the supernatant. The supernatant and biomass were kept for further analysis.

### 2.4. Study of the Effect of Xylose Concentration

Different xylose concentrations (5, 10, 20, 30, 40, 50, 60, 70, 80 g L^−1^) were used to determine their effect on protein content and biomass production in Erlenmeyer flasks with basal medium. Before autoclave, initial pH was adjusted to 5.0. The biomass that was produced, its protein content and the xylose consumption were measured after 8 days.

### 2.5. Study on the Effect of Glucose/Xylose Mixtures

To investigate the effect of different glucose/xylose mixtures on protein and biomass production of *P. ostreatus* LGAM 1123 five different ratios of these sugars were used. 50 g L^−1^ of glucose and 0 g L^−1^ xylose (50G0X), 35 g L^−1^ of glucose and 15 g L^−1^ xylose (35G15X), 25 g L^−1^ of glucose and 25 g L^−1^ xylose (25G25X), 15 g L^−1^ of glucose and 35 g L^−1^ xylose (15G35X), 0 g L^−1^ of glucose and 50 g L^−1^ xylose (0G50X). Every two days, samples were withdrawn from cultivation for the estimation of biomass, sugars and protein content. Cultivation lasted for 12 days, until sugars were totally consumed.

### 2.6. Cultivation on Aspen Hydrolysate

The aspen hydrolysate was generated after steam explosion pretreatment of aspen wood chips, which was performed in 40 L reactor at 175 °C, with 25% *w*/*w* total solids for 10 min. H_2_SO_4_ was added to a final concentration of 1% *w*/*w*. The generated slurry was enzymatically hydrolyzed using an enzyme loading of 0.1 g/of enzyme preparation per g of DM in the slurry. Cellic CTec II (Novozymes, Bagsværd, Denmark) was used as the enzyme cocktail. Hydrolysis was performed at 50 °C, at pH 5, with 8% suspended solids.

Prior to fermentations, the hydrolysate was diluted 2 times and minerals and yeast extract were added to the cultivation medium to match growth conditions of *P. ostreatus* in previous experiments (Section 2.3). 4 L STR bioreactors (Belach Bioteknik, Skogås, Sweden) with a working volume of 3 L were used for the cultivation. An inoculum of 5% (*v*/*v*) of the working volume was added in the bioreactor. Cultivation temperature and pH were adjusted to 28 °C and 5, respectively. The agitation speed varied from 200 to 800 rpm depending on the oxygen demand and viscosity increase in the culture. At specific time intervals, samples were withdrawn for biomass and protein estimation.

### 2.7. Biomass and Reducing Sugars Determination

Five milliliters of fungal cultures were used for the quantification of biomass. The biomass was separated from supernatant using centrifugation (4000 rpm, 10 min) and the supernatant was kept for the reducing sugar analysis. The biomass was washed 3 times, and the samples were freeze-dried. The dry biomass was weighed using an analytical scale (Ohaus PX323 Pioneer analytical balance). Reducing sugar concentrations were determined colorimetrically from the supernatant using DNSA method as described by Miller [23]. Specifically, 0.5 mL of supernatant was mixed with 0.5 mL of DNS reagent. After boiling the mixture for 5 min, 4 mL of milli-Q water was added and absorbance was measured at 540 nm using a UV-visible spectrophotometer (JASCO, V-730). The concentration was estimated from absorbance using a standard curve for glucose or xylose. To determine sugars separately, glucose oxidase from *Aspergillus niger* (Sigma-Aldrich (St. Louis, MO, USA)) was used to transform glucose to D-glucono-δ-lactone, which does not react with the DNSA reagent, enabling us to determine only xylose concentration in glucose/xylose cultivation mixtures.

Biomass yield was estimated from biomass production and consumed sugars according to the equation:

Biomass yield % (g of biomass produced/100 g of sugars consumed) = Biomass production (g L^−1^)/Sugars consumed (g L^−1^) ∗ 100 (1)

### 2.8. Protein Estimation

Total protein content from dry biomass was estimated using the Dumas method, as previously described [11], after total nitrogen content had been measured. Protein production was estimated from protein content and biomass production according to the equation:

Protein production (g L^−1^) = Protein content (% dry weight) ∗ Biomass production (g L^−1^)/100 (2)

Protein yield was estimated from protein production in dry weight and consumed sugars according to the equation:

Protein yield % (g of protein produced in dry weight/100 g of sugars consumed) = Protein production (g L^−1^)/Sugars consumed (g L^−1^) ∗ 100 (3)

### 2.9. Amino Acid Analysis

For amino acid analysis a chemical hydrolysis of biomass was conducted using 6 M HCL as previously described [11] The hydrolyzed sample was kept at −20 °C for amino acid analysis [24,25].

For amino acid analysis, dabsyl chloride was used as a derivatization reagent as performed according to Ribeiro et al. [26]. The above derivatives were separated on an HPLC unit (Shimadzu, Kyoto, Japan) equipped with a photodiode array detector and a reversed-phase C18 column (μBondapack, Waters, Wexford, Ireland) with dimensions of 3.9 × 300 mm, 10 μm particle size, and 125 Å pore size. The HPLC method was the same used by Ribeiro et al. [26] Detection was achieved at 461 nm. Amino acid quantification was accomplished by area recorded in the chromatograms relative to external amino acid standards [26].

### 2.10. Aspen Hydrolysate Analysis

Levulinic acid, HMF, furfural, acetic acid, formic acid and ethanol were analyzed using an HPLC unit (Agilent technologies 1260 Infinity system) equipped with a refractive index (RI) detector, with a Biorad Aminex HPX-87H 300 × 7.8 mm column. The elution was performed at a flow rate of 0.6 mL/min with 19 mM H_2_SO_4_ at a temperature (oven and detector) of 55 °C.

Glucose, xylose, mannose, cellubiose, arabinose, galactose in aspen hydrolysate were analyzed using an HPLC unit (Agilent technologies 1260 Infinity system) equipped with RI detector using a Biorad HPX-87P 300 × 7.8 mm column and a Biorad micro-guard column carbo P cartridge 30 × 4.6 mm. The analysis was performed using milli-Q water as the eluent and a flow rate of 0.6 mL/min. The oven temperature was set at 75 °C, and the detector temperature was 55 °C.

### 2.11. Principal Component Analysis (PCA) and Statistical Analysis

PCA was performed on amino acids concentrations to determine potential groupings or clustering as well as eventual correlation with the experimental conditions for subsequent analysis. The first PC is defined as that giving the largest contribution to the respective PCA of linear relationships exhibited in the data. The second component may be considered as the second-best linear combination of variables that accounts for the maximum possible residual variance after the effect of the first component is removed from the data [27]. Amino acid concentrations were set as the variables, and the glucose/xylose ratios were set as the primary ID observations (model type: PCA-X with 5 observations and 17 variables). The software SIMCA 17 (SIMCA^®^, Sartorius, Göttingen, Germany) was used for the PC analysis.

All experiments were conducted in triplicate. The values were presented as mean ± standard deviation. Statistical analysis was conducted using one-way analysis of variance (ANOVA) and Tukey’s multiple range test, with *p* values <0.05 being regarded as significant (IBM SPSS statistics, version 28.0.1.0, IBM Corporation, New York, USA). PCA was conducted using SIMCA 17 (SIMCA^®^, Sartorius).

## 3. Results

### 3.1. Effect of Xylose Concentration

The effect of xylose concentration was studied as described in Section 2.3. Cultivation lasted for 8 days, during which the xylose had been totally consumed, except for the cases of initial xylose concentrations of 60 g L^−1^ and 80 g L^−1^ (where xylose consumption after 8 days of cultivation was 93.7 ± 0.4% and 71.2 ± 6.7%, respectively). As we can see from Table 1, the best biomass production was achieved at a xylose concentration of 60 g L^−1^, which reached a value of 20.4 ± 0.3 g L^−1^, showing a difference that was not statistically significant (*p* > 0.05) with the respective values (20.4 ± 2.9 and 19.4 ± 0.4 g L^−1^) when concentrations of 80 g L^−1^ and 50 g L^−1^ were used. Protein content was reached a maximum for 10 g L^−1^ xylose with a value of 42.9 ± 0.8%, while xylose concentrations of 20 and 30 g L^−1^ were followed by different protein content values (42.6 ± 0.4 and 39.3 ± 2.1%, respectively) that were not significant different (*p* > 0.05).

### 3.2. Effect of Glucose/Xylose Mixtures

**Figure 1:**
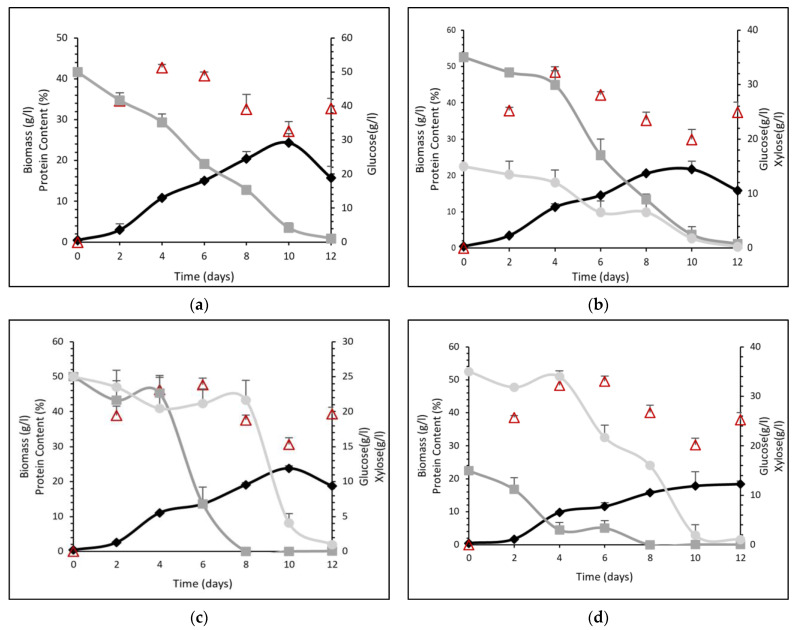

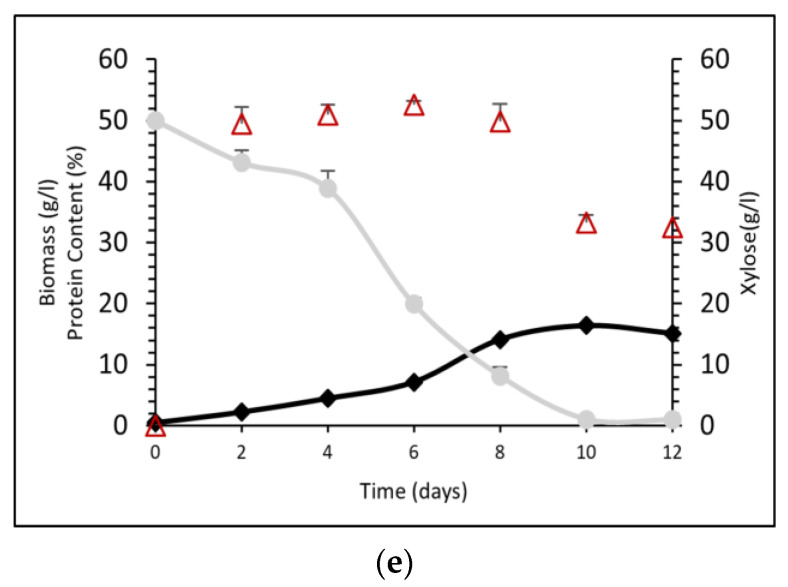
Growth curves (lines with rhombus symbols), sugars consumption (glucose: line with square symbol, xylose: line with circle symbol) and protein content (triangle symbols) in different time intervals for *P. ostreatus* LGAM 1123 cultivation on different mixtures of glucose and xylose. (**a**) 50G0X, (**b**) 35G15X, (**c**) 25G25X, (**d**) 15G35X, (**e**) 0G50X.

As described in Section 2.4, the cultivation of *P. ostreatus* LGAM 1123 lasted 12 days. When only glucose was used as the sugar in the culture medium (mixture 50G0X), biomass production was reached a maximum on the 10th day of cultivation, reaching a value of 24.3 ± 2.3 g L^−1^, followed by a death phase (Figure 1a). Glucose was almost totally consumed after 12 days of cultivation with only 1.0 ± 0.07 g L^−1^ remaining in the cultivation medium. Protein content was maximum on the 4th day of cultivation with the value of 42.7 ± 1.6%. Protein content was stable until the 6th day of cultivation and after that decreased up to a value of 32.8 ± 0.3% on the last day of cultivation.

When a mixture of glucose and xylose (35G15X) was used as a carbon source in the culture medium, biomass reached its maximum value on 10th day of cultivation (21.7 ± 2.2 g L^−1^), followed by a death phase (Figure 1b). Concerning sugar consumption, a delay was observed for the first 4 days. After that, glucose seemed to be consumed with a faster rate compared to xylose. After 12 days of cultivation, glucose and xylose were almost totally consumed with only 0.7 ± 0.2 g L^−1^ and 0.3 ± 0.07 g L^−1^ remaining in the cultivation medium, respectively. Protein content reached its maximum value (48.4 ± 0.1%) on the 4th day of cultivation, keeping a stable value until day 6, and after that, a decrease was observed. The final protein content on the 12th day of cultivation was 37.4 ± 0.2%.

In the case of mixture 25G25X, maximum biomass production (23.8 ± 0.7 g L^−1^), was also observed on the 10th day of cultivation, followed by a death phase (Figure 1c). Regarding sugar consumption, it is obvious that only after glucose was almost totally consumed after 8 days of cultivation did xylose start to be consumed. The remaining sugar concentration for glucose and xylose after 10 days were 0.04 ± 0.01 g L^−1^ and 1.0 ± 0.2 g L^−1^, respectively. Protein content was maximum at the 6th day of cultivation reaching a value of 47.6 ± 3.9%. Protein content was stable until the 8th day of cultivation, and after that, a decrease up to a value of 30.6 ± 3.9% at the last day of cultivation was observed.

When mixture 15G35X was used, biomass reached its maximum value (18.5 ± 0.8 g L^−1^) on the 12th day of cultivation (Figure 1d). As we can observe, in the first 4 days, there was a delay in xylose consumption, whereas glucose was consumed, reaching 3.0 ± 1.5 g L^−1^. After that, xylose started to be consumed with a faster rate, reaching 1.1 ± 0.1 g L^−1^ at the last day of cultivation. The protein content reached a maximum on the 6th day of cultivation, reaching a value of 49.6 ± 3.6%. After that a decrease was observed. The final protein content on the 12th day of cultivation was 37.9 ± 0.3%.

Finally, the last cultivation medium studied was that which only contained xylose without the presence of any glucose (0G50X). Biomass production was maximum after 10 days of cultivation reaching a value of 16.4 ± 0.3 g L^−1^. Concerning xylose consumption, we can observe a delay for the first 4 days of cultivation, while after that, the consumption was rapid. The final xylose concentration was 0.9 ± 0.02 g L^−1^. The protein content reached a maximum after 6 days of cultivation reaching, a value of 52.6 ± 1.1%, whereas the final protein content on the 12th day of cultivation was 32.6 ± 0.1%.

Despite leading to less biomass production, xylose led to a higher protein content of the mycelium compared to glucose. When the nutrient medium contained a mixture of glucose and xylose, a high concentration of mycelium with a high protein content was produced.

**Figure 2:**
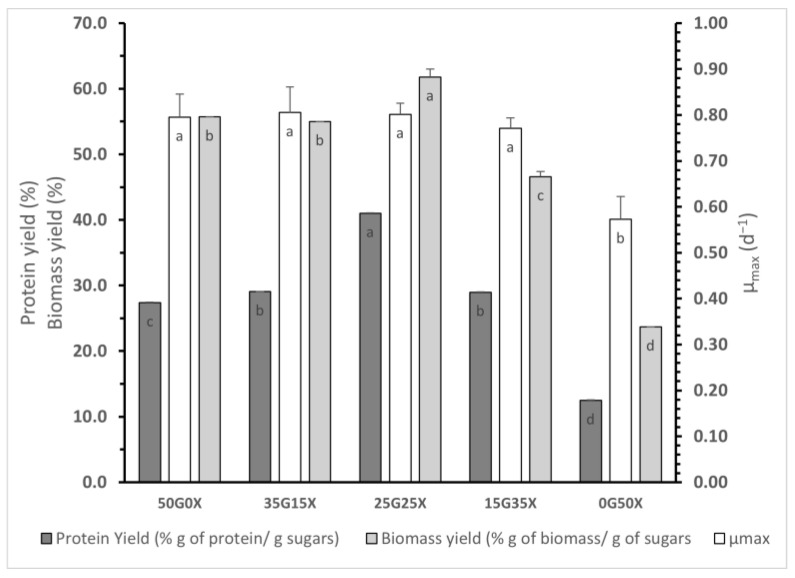
Maximum protein yields, biomass yields and specific growth rates (μ) for *P. ostreatus* LGAM 1123 cultivation on different glucose/xylose mixtures. Different letters indicate a significant difference (*p* ≤ 0.05) in Tukey’s multiple range test.

In Figure 2, the maximum protein yields, biomass yields and specific growth rates (μ) for submerged cultivation of *P. ostreatus* LGAM 1123 on different glucose/xylose mixtures are depicted. The optimum protein yield was observed for mixture 25G25X reaching a value of 41.0 ± 0.07%, while mixtures 35G15X and 15G35X followed leading to values of 29.1 ± 0.0 and 29.0 ± 0.02%, respectively (*p* > 0.05). In a similar way, maximum biomass yield was observed for mixture 25G25X (61.8 ± 1.2%), while 50G0X and 35G15X mixtures followed with values of, 55.7 ± 0.05% and 55 ± 0.03%, respectively. Concerning specific growth rates (μ), when xylose was used as the only sugar in culture medium, a lower value was observed. Contrarily, the presence of glucose in the medium seemed to result in higher specific growth rates. More specifically, a value of 0.8 ± 0.15 d^−1^ was achieved when only glucose was used as a carbon source in the medium, while similar values were also found when a glucose/xylose mixture was used instead. Specific growth rate values found for mixtures 35G15X, 25G25X and 15G35X were not significantly different with that of mixture 50G0X (*p* > 0.05).

### 3.3. Amino Acids Analysis for P. ostreatus LGAM 1123 Cultivation on Different Glucose/Xylose Mixtures

As it can be seen in Table 2, 17 amino acids were detected from *P. ostreatus* LGAM 1123 biomass, including 7 of 9 essential amino acids (except for histidine (His) and threonine (Thr)). For all culture mediums studied, the most abundant amino acids observed were glycine (Gly) and proline (Pro), and leucine (Leu), valine (Val), phenylalanine (Phe), and arginine (Arg) were also detected in large amounts. Regarding essential amino acids, Leu was the most abundant one, followed by Val and Phe, while lysine (Lys) was the last in a descending rank order. Leu reached a value of 103.5 ± 3.5 mg/g protein in the case of mixture 50G0X, while lower glucose concentrations in the growth medium led to lower Leu concentrations. Similarly, a higher concentration (82.4 ± 2.6 mg/g protein) was found for Val when glucose was used as a sole sugar (mixture 50G0X) in comparison to that which was determined (69.3 ± 0.2 mg/g protein) when only xylose was used in the growth medium (0G50X). On the contrary, a higher content of Phe was observed for the medium with the higher xylose concentration among different combinations of glucose and xylose concentrations (93.7 ± 7.7 mg/g protein for 0G50X compared to 66.7 ± 0.1 mg/g protein for 50G0X). A similar behavior was also observed for isoleucine (Ile). Its content seemed to increase with the increase in the concentration of xylose in the growth medium ranging from 47.1 ± 2.3 mg/g protein for mixture 50G0X to 54.0 ± 1.9 mg/g protein for mixture 0G50X. Concerning the other essential amino acids, the content of tryptophan (Trp) did not seem to be substantially affected by the glucose to xylose ratio of the culture medium, while methionine (Met) presented higher concentration in the 25G25X mixture (12 ± 0.6 mg/g protein). Finally, the Lys concentration values were found to be similar for all the cultivation conditions used. It should be noted that the amino acid composition values found for *P. ostreatus* LGAM 1123 biomass for all growth mediums tested in this work is over the minimum recommended patterns for Val, Ile, Leu, and aromatic amino acids (AAA), according to the report of FAO Expert Consultation for Dietary Protein Quality Evaluation in Human Nutrition [28].

**Figure 3:**
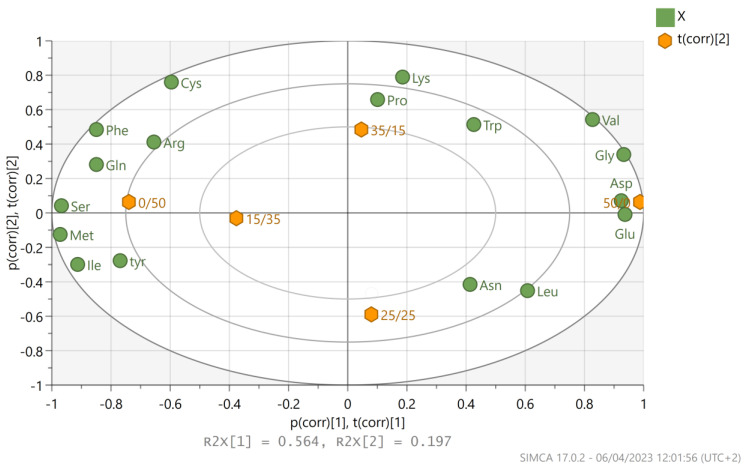
PCA Analysis biplot (combination of scores and loadings plots) for amino acid composition on different glucose/xylose mixtures depicting the corresponding loading (green circles) and scores (orange rhombus) plots that establish the relative importance of each variable (symbols: 50/0 is mixture 50G0X, 0/50 is mixture 0G50X, 35/15 is mixture 35G15X, 25/25 is mixture 25G25X).

To obtain an overview of the effects of glucose and xylose contained in the growth medium on the protein amino acid composition of mycelium, we subjected the relative changes in amino acid composition at different mixtures of glucose and xylose to principal component analysis (PCA) (Figure 3; see also Supplementary Material S1 for full data from PCA analysis). In Figure S1, the corresponding loading (on the right) and scores (on the left) plots that establish the relative importance of each variable (amino acids concentrations) are shown. The first PC, which explains 56,4% of the variance, is associated with the presence or absence of glucose and xylose. As it can be seen, it correlates positively with the amino acids Trp, Gly, asparagine (Asn), aspartic acid (Asp), Leu, and glutamic acid (Glu), and negatively with Arg, serine (Ser), Met, Ile, cysteine (Cys), and tyrosine (Tyr), while Pro and Lys do not have high weight on this component, showing that the presence of glucose (or the absence of xylose) favors the production of Val, Gly, Asp and Glu, while the absense of glucose favors the formation of Ser, Met, and Ile. The second PC (explaining 19.7% of the variance) correlates negatively with Asn, Leu, Ile and Tyr, while Met, Ser, Asp and Glu do not have high weight on this component. From Figure 3, a clear clustering can be identified across the PC1, which correlates the presence of Asp, Glu, Gly, and Val with the presence of glucose in the media (right part of the graph), or the presence of Arg, Cys, Phe, Gln, Tyr, Ser, Met and Ile with the presence of xylose (left part of the graph). These results are in accordance with Table 2.

### 3.4. Protein Production by P. ostreatus LGAM 1123 in a 4 L Stirred-Tank Bioreactor Using Aspen Hydrolysate

**Figure 4:**
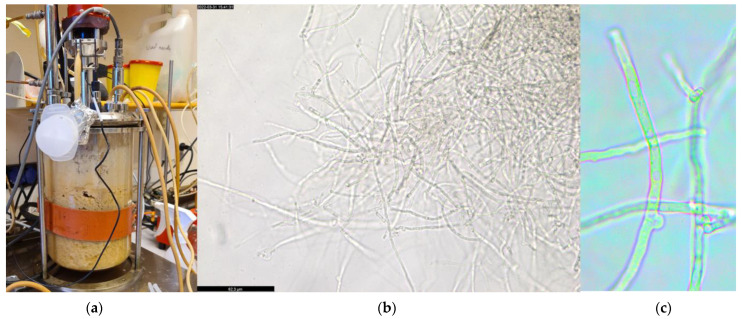
(**a**) Cultivation of *P. ostreatus* LGAM 1123 in a 4 L STR bioreactor (Belach Bioteknik, Sweden) using aspen hydrolysate, (**b**) *P. ostreatus* LGAM 1123 mycelia photography by an optical microscopy (magnitude 40×), (**c**) 100× magnitude observation of *P. ostreatus* LGAM 1123 mycelia.

To assess the potential of *P. ostreatus* LGAM 1123 for industrial applications, aspen hydrolysate composed of glucose and xylose as its main sugars was used as the carbon source for the cultivation of the fungus in a 4 L STR bioreactor (Figure 4). Before cultivation, aspen hydrolysate was analyzed for possible inhibitors. The composition of aspen hydrolysate is shown in Table 3.

**Figure 5:**
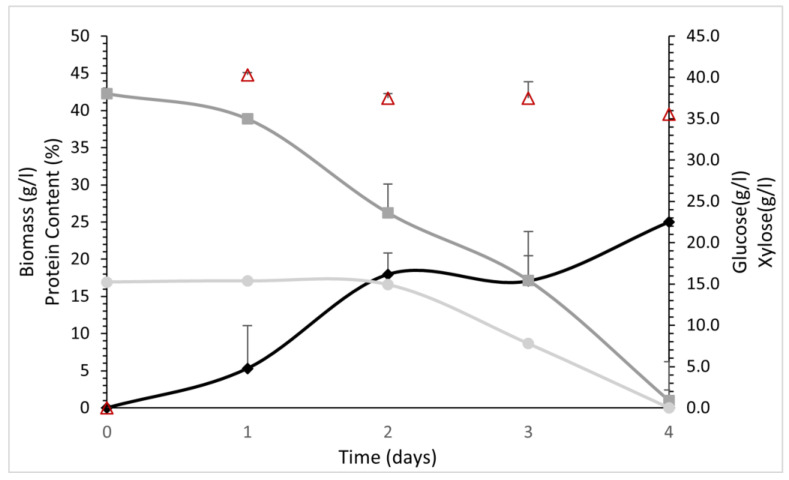
Growth curves (lines with rhombus symbols), sugar consumption (glucose: line with square symbol, xylose: line with circle symbol) and protein content (triangle symbols) at different time intervals for *P. ostreatus* LGAM 1123 cultivation on aspen hydrolysate.

The hydrolysate was diluted two times in order to be used as cultivation substrate. Consequently, the initial glucose and xylose concentrations were 38 g L^−1^, and 15 g L^−1^, respectively. The pH was kept stable at 5 for all the fermentation process. As we can see in Figure 5, only glucose consumption was observed for the first two days of cultivation (glucose concentration 23.6 ± 3.5 g L^−1^ at this point), whereas xylose consumption started after this point. Glucose and xylose were totally consumed after 4 days of cultivation. Fungal biomass reached 25.0 ± 3.4 g L^−1^ at the end of the cultivation, while biomass yield reached a value of 46.9 ± 0.03%. The specific growth rate (μ) was 1.8 ± 0.4 d^−1^. The protein content was almost stable during the fermentation, reaching a maximum value of 44.0 ± 0.3% after 2 days of cultivation and a protein yield of 54.5 ± 0.5%.

## 4. Discussion

*P. ostreatus* LGAM 1123 has been previously cultivated, as described by Bakratsas et al. 2023, in glucose-based medium [11]. The optimization process revealed that protein content was at its maximum with a value of 49.0% at 42.7 g L^−1^ glucose and 17.8 g L^−1^ yeast extract. Biomass production reached 28.9 g L^−1^ when concentrations of glucose and yeast extract were 54.14 g L^−1^ and 17 g L^−1^, respectively. Maximum protein production (13.6 g L^−1^) was achieved when glucose and yeast extract concentrations were 54.14 g L^−1^ and 18.25 g L^−1^, respectively. According to these results, glucose concentrations from 0 g L^−1^ to 50 g L^−1^ were used in this work. Concerning xylose concentration, a study of the effect of xylose concentration on protein and biomass production was conducted. When the xylose concentration in the growth medium increased up to 50 g L^−1^, an increase in biomass production was observed, reaching a value of 19.4 ± 0.4 g L^−1^, while at larger xylose concentrations, a plateau was observed (Table 1). This result is in accordance with Papaspyridi et al. 2010 and Songulashvili et al., 2008 [29,30]. More specifically, in an optimization study of biomass production by *P. ostreatus* cultivation using xylose as a carbon source and corn steep liquor as a nitrogen source, biomass increased with the increase in xylose concentration up to 57 g L^−1^, whereas further increase in xylose concentration had a negative impact on biomass production [29]. Additionally, in a study of submerged cultivation of *Ganoderma lucidum* in xylose medium, an increase in xylose in growth medium from 0.5% to 1% led to an increase in biomass, whereas further increase in xylose concentration led only to slight modifications of biomass production [30]. Increasing the concentration of xylose in the growth medium up to 30 g L^−1^ leads to an increase in the biomass protein content, reaching a maximum value of 42.6 ± 0.4%. At higher concentration levels of initial xylose in the culture medium, the protein content decreases. An explanation to this is that when biomass concentration is increased, the culture oxygen demand reaches a level that cannot be met, and this is reflected in the lower protein content. This result is in accordance with a previous study by our group with *P. ostreatus* LGAM 1123. The cultivation of this strain showed a similar protein content decrease when the initial glucose concentration was higher than 30 g L^−1^ [11].

All the above results indicate that *P. ostreatus* LGAM 1123 could be cultivated both in a C-6 (glucose)- and in a C-5 (xylose)-sugar-containing medium, producing biomass with high protein content. Cultivation in mixtures of glucose and xylose has potential as these sugars are the principal ones in lignocellulosic biomass produced by chemical or enzymatic hydrolysis [14]. To our knowledge, *P. ostreatus* has not previously been cultivated in mixtures of glucose and xylose. According to our results, glucose favored biomass production, reaching a value of 24.3 ± 2.3 g L^−1^ in the case of 50G0X culture mixture, whereas the production decreased when lower glucose concentrations were supplemented in the medium (16.4 ± 0.3 g L^−1^ at 0G50X). This could possibly be explained by the slower xylose consumption rates and the lag phase observed when xylose or a glucose/xylose mixture was used compared to glucose consumption rates. In contrast, taking into consideration sugar consumption, glucose/xylose mixtures seemed to favor biomass yield, reaching a value of 61.8 ± 1.2% in the case of 25G25X mixture, whereas using xylose as a sole source of carbon led to the lowest biomass yield (23.7 ± 0.02%). A similar study for lipid production by *Mortierella isabellina* using glucose/xylose mixtures in growth medium has confirmed that the glucose uptake rate is higher than that of xylose. In addition, biomass production was reduced as growth medium was supplemented by lower concentrations of glucose, ranging from 29.5 g L^−1^ for a medium containing only glucose as a sugar to 20.7 g L^−1^ for a xylose-containing medium. The highest biomass yield was achieved for a mixture of 60 g L^−1^ glucose and 20 g L^−1^ xylose [15]. The higher glucose uptake rate and biomass production in a glucose-based medium in contrast to a xylose or a glucose/xylose mixture-based medium was confirmed in another study on lipid production by *Thamnidium elegans*. It has been shown that a maximum biomass yield equal to 31.9% was achieved in a growth medium containing 100 g L^−1^ glucose and 0 g L^−1^ xylose, whereas biomass yield reached only 21.4% in a medium with 0 g L^−1^ glucose and 100 g L^−1^ xylose [14]. Concerning protein content, we can see that, in all conditions, protein content was higher in the exponential phase of cultivation, whereas in the stationary phase, protein synthesis rate was lower. This could be explained by the fact that after exponential phase, cells change their metabolism to adapt to the new environment where the oxygen and carbon sources are decreased, toxic by-products are increased, and growth is suspended by the density of neighboring cells [31]. Xylose seems to produce biomass with higher % protein content, reaching a value of 52.6 ± 1.1%, compared to a 100% glucose-containing medium, reaching a protein content of 42.7 ± 1.6%. A higher protein content (19.4 ± 0.5%) of biomass produced in a xylose-containing medium in comparison to a glucose-containing one (14.0 ± 0.1%) was also confirmed by a study of submerged cultivation of *Pleurotus pulmonarius* [26]; however, taking into consideration sugar consumption, protein production yields seem to be higher in glucose/xylose mixtures in contrast to sole glucose- or sole xylose-containing mediums. Specifically, the 25G25X mixture produced 41 ± 0.7% g of protein produced/g of sugars consumed, whereas the 35G15X and 15G35X mixtures followed with respective values of 29 ± 0.0% and 29 ± 0.02% g of protein produced/g of sugars consumed (*p* > 0.05). A 100% xylose-containing medium led to the lowest protein production yield (13 ± 0.07% g of protein produced/g of sugars consumed). Papaspyridi et al. [29] reported a lower protein production yield of 8.9% g of protein produced/g of sugars consumed for *P. ostreatus* cultivation in a medium containing 57 g L^−1^ xylose, whereas a protein yield value closer to our results (equal to 15%) was observed for *Pleurotus sajor-caju* cultivation on corn stover [29,32]. Smiderle et al., 2012 [25] reported similar results to ours, with a protein yield higher in a glucose-based medium in contrast to a xylose-based one (4% and 0.4% in glucose- and xylose-containing medium, respectively) [25].

Specific growth rate seems to be favored by glucose existence in the cultivation medium with the maximum value achieved in the 35G15X mixture, reaching a value of 0.81 d^−1^. The lowest specific growth rate was observed when the 0G50X mixture was used. Similar growth rates were reported for cultivation of *P. ostreatus* in a medium of 40 g L^−1^ glucose and 5 g L^−1^ yeast extract (0.87 d^−1^), as well as for cultivation of *Pleurotus sajor caju* on corn stover (0.86 d^−1^) [32,33].

Although, yields for *P. ostreatus* have not been calculated for submerged aerobic growth, approximate estimations could be made from studies on yeasts, where yields about 0.52 g biomass (g glucose)^−1^ have been reported. However, it should be considered that theoretical yields for aerobic growth may vary significantly for different microorganisms [31]. A comparison between theoretical and experimental aerobic growth yields is complicated by the fact that growth yields in aerobic organisms depend on both the energy requirement for biosynthesis (YATP) and the ATP yield of respiration (P/O-ratio). Moreover, many assumptions and simplifications are made in the theoretical calculations (energy needed for protein formation, cell maintenance energy) [31]. An eventual difference from the theoretical yield could be explained not only by the above-mentioned assumptions used in most theoretical calculations, but also by the lack of optimum conditions during the cultivations. The yields obtained here during the submerged cultivation of *P. ostreatus* reached 0.62 ± 0.012 g of biomass (g glucose)^−1^ [30,31,32,33]. In addition, summarizing the above results, we could conclude that a mixture of glucose and xylose in the basal growth medium of *P. ostreatus* LGAM 1123, could enhance protein yield and growth rate. Cultivation in a side stream of glucose/xylose mixtures could be the solution for lower production costs, and circular economy reinforcement. As we can see from Section 3.4, *P. ostreatus* LGAM 1123 cultivation in a 4 L stirred-tank bioreactor using aspen hydrolysate was achieved. The growth of strain was rapid with a maximum biomass production of 25.0 ± 3.4 g L^−1^ and a biomass yield of 46.9 ± 0.03%. The maximum specific growth rate and protein yield achieved in this study were 1.8 ± 0.4 d^−1^ and 54.5 ± 0.5%, respectively. In a study of submerged cultivation of *P. ostreatus ATHUM* 4438 in a 17 L (working volume) stirred-tank bioreactor with 57 g L^−1^ xylose, a higher biomass yield (68.7%) and lower specific growth rate (1.22 d^−1^) and protein yield (8.9%) were observed [29]. A study of submerged cultivation of *Thamnidium elegans* in a 3 L bioreactor with a working volume of 1.5 L with 100 g L^−1^ glucose resulted in a higher biomass production of 30.1 g L^−1^ but in a lower biomass yield [14].

Treatment of lignocellulosic biomass to produce hydrolysates offers numerous benefits; although, a drawback is the chemical inhibitors generated during the pretreatment process [34]. In this work the concentrations of possible inhibitors included in the aspen hydrolysate are presented on Table 3. The successful growth of the *P. ostreatus* LGAM 1123 in the aspen hydrolysate has shown that the concentrations of substances, such as levulinic acid, HMF, furfural, acetic acid, formic acid and ethanol, were not adequate to inhibit fungus growth. The concentrations of HMF, furfural and acetic acid in our work were lower compared to those respectively reported by Vanmarcke et al. for an aspen hardwood hydrolysate, while concentrations of levulinic acid and formic acid were found to be higher in our hydrolysate [35]. The growth of fungi in the presence of higher concentrations of inhibitors formed during pretreatment of lignocellulosic material was also confirmed by a study on the cultivation of *Fusarium oxysporum* [36]. Finally, the concentration of these inhibitors is so low that they could not be a problem for the mycoprotein, in which only traces of these substances could be detected. Tolerable daily intakes of such substances according to European Commission are 0.54 mg/day for HMF and furfural, 0–3 mg/kg body weight for formic acid and 1–2.1 g/day for acetic acid [37,38,39,40]. Generally, food-grade substrates that can be used for mycoprotein production are limited to food crops and starch-derived glucose. The next step for mycoprotein production industry is to use non-crop feedstocks, exhibiting minimum land needs for their production and reduced carbon footprint (side streams from food and beverages industries, forestry and agro-industrial residuals forms [41,42]; however, technoeconomic challenges associated with the increased number of unit operations that are required when dealing with complex substrates have to be tackled to make these processes economically viable. Apart from the decrease in energy requirements in every bioconversion step, a significant boost to this technology could be given by an eventual considerable increase in biomass and protein yields.

Amino acid analysis of the fungus protein derived from *P. ostreatus* LGAM 1123 biomass was conducted for all glucose/xylose mixtures used in order to estimate the nutritional value of protein and evaluate differences between the cultivation conditions. Fungi can catabolize glucose into amino acids starting with glycolysis in order to convert glucose to pyruvate and, after that, using tricarboxylic acid cycle (TCA cycle). For the utilization of xylose, three pathways have already been proposed. One in which xylulose is produced from xylitol with NAD+-dependent xylitol dehydrogenase (XDH), and before this step, xylose has already been converted into xylitol by NAD(P) H-dependent xylose reductase (XR). The other way is using xylose isomerase (XI) to directly convert xylose to xylulose. After this step xylulose kinase is used to convert xylulose to xylulose-5-phosphate. The next step is one that connects xylose metabolism with the glucose one, using the pentose phosphate pathway (PPP), which leads to Fructose 6-phosphate or Glycerol-3-phosphate [43]. A new pathway that connects xylose directly to the TCA cycle was proposed for ascomycetes *Myceliophthora thermophila* [44]. In this pathway, D-xylose dehydrogenase (XDH) was used to convert D-xylose to D-xylonolactone. D-xylonolactonase (XL) hydrolyzes D-xylonolactone into D-xylonate. D-xylonate dehydratase (XD) dehydrates D-xylonate to 2-keto-3-deoxy-d-xylonate. 2-keto-3-deoxy-d-xylonate dehydratase converts 2-keto-3-deoxy-d-xylonate to 2,5-Dioxopentanoate. In the last step 2-ketoglutarate semialdehyde dehydrogenase converts 2,5-Dioxopentanoate to a-Ketoglutarate [44]. Amino acids in *P. ostreatus* could be divided into those produced by glycolysis, such as Ala, Gly, Val, Cys, Phe, Tyr, Ser, Leu, Ile and Trp; those produced by the TCA cycle, such as Asp, Glu and Lys; those produced from glutamic acid, such as Arg and Pro; and those produced from aspartic acid, including Cys, Met, Thr, Asn, and His [45]. From our results, the most abundant amino acids for all studied conditions were Gly and Pro, whereas Leu, Val, Phe, and Arg were also detected in large amounts. These results were also confirmed in a previous study conducted by our group on single-cell protein production in submerged cultivation of the same strain [11]. In comparison, the amino acid analysis conducted in a study of submerged cultivation of *Pleurotus pulmonarius*, revealed that amino acids with the highest contents were Phe, Asp, Glu, and Pro [25]. In contrast, amino acids with the highest contents from *Cordyceps militaris* mycelium were Thr, Gly, and Glu [46]. PCA analysis revealed a clear clustering, which correlates the overexpression of Asp and Glu (amino acids responsible for umami taste), Val (an essential amino acid) and Gly, with the presence of glucose (or the absence of xylose). In a similar way the presence of xylose (or the absence of glucose) in the medium was correlated with Ser, Tyr, Gln, Arg, Cys, as well as the essential amino acids Met, Ile and Phe. Therefore, differences observed in the amino acid composition of the protein produced using glucose or xylose therefore cannot generally be explained by differences in anabolic pathways; however, they could be explained by the differential regulation of these anabolic pathways due to differences in the bioenergetic of glucose and xylose catabolism, such as different uptake mechanisms, and ATP and redox requirements. Further experiments using other sugar combinations (xylose–mannose, glucose–galactose, glucose–mannose) as carbon sources could reveal the nature of this correlation and show if it is mostly associated with the absence or the presence of a certain sugar. To our knowledge, there are no other studies conducting PCA analysis to cluster amino acids with the glucose and xylose metabolism of fungi. One study conducted for *P. ostreatus* cultivation in black poplar wood logs (WS) and lignocellulosic by-products (LcS) revealed that different amino acids were expressed in the presence of different substrates [47]. Another study concerning the different free amino acids expression in three *Pleurotus* strains has been conducted. Smiderle et al., who studied the submerged cultivation of *Pleurotus pulmonarius*, confirmed that Leu, Asp and Glu are favored by glucose, while Met, Ile and Gln are higher in a xylose-containing medium. In contrast to our results, Phe presented a higher concentration in the case of a glucose medium [25]. The amino acid composition values found in this work for biomass protein produced are much higher than the recommended patterns for Val, Leu, and AAA. In the case of Ile, its content determined in the produced protein was adequate for children, adolescents and adults. *P. ostreatus* protein is not adequate for Lys and sulfur amino acids (SAA) recommended scores in human diet [29].

## 5. Conclusions

*P. ostreatus* biomass produced by submerged fermentation in a batch-stirred tank bioreactor using agro-industrial hydrolysate could be used as mycoprotein due to its high protein content 52.6 ± 1.1% and a protein yield of 54.5 ± 0.5% (g of protein produced/g of sugars consumed). In addition, amino acid analysis of the biomass produced resulted in higher concentrations of most of the essential amino acids compared to the recommended values in human nutrition according to FAO Expert Consultation for Dietary Protein Quality Evaluation (2013). PCA analysis revealed a clear clustering, which correlates the presence of certain amino acids with the presence of glucose or xylose. This result is a clear indication that different enzymes are produced and that different pathways are activated in each case. Proteomics combined with metabolomic analysis would further elucidate this topic for *P. ostreatus* cultivation and will be the objective of future research. Although high specific growth rates (0.81 d^−1^ in Erlenmeyer flasks and 1.8 ± 0.4 d^−1^ in bioreactor) and high protein content (44.0 ± 0.3%) were achieved for submerged cultivation of *P. ostreatus* in glucose/xylose mixtures, technoeconomical and sustainability challenges associated with mycoprotein production need to be tackled to become an economically viable option at a commercial scale.

## Figures and Tables

**Table 1:** Total protein content, biomass production and xylose consumption after 8 days of *P. ostreatus* LGAM 1123 cultivation on different xylose concentrations. No common letters in each column indicate significant difference (*p* ≤ 0.05) in Tukey’s multiple range test.

Xylose Concentration (g L^−1^)	Biomass (g L^−1^)	Xylose Consumption (%)	Total Protein Content (%)
5	2.5 ± 0.2 ^g^	100 ± 0.0 ^a^	10.7 ± 1.5 ^g^
10	6.4 ± 0.4 ^f^	100 ± 0.0 ^a^	42.9 ± 0.8 ^a^
20	9.7 ± 0.0 ^e^	100 ± 0.0 ^a^	42.6 ± 0.4 ^ab^
30	13.3 ± 0.6 ^d^	100 ± 0.0 ^a^	39.3 ± 2.1 ^ab^
40	16.2 ± 1.5 ^c^	100 ± 0.0 ^a^	32.2 ± 1.8 ^cdef^
50	19.4 ± 0.4 ^ab^	100 ± 0.0 ^a^	33.6 ± 1.2 ^cde^
60	20.4 ± 0.3 ^a^	93.7 ± 0.4 ^b^	30.7 ± 2.1 ^ef^
80	20.4 ± 2.9 ^ab^	71.2 ± 6.7 ^c^	37.5 ± 7.8 ^abcd^

**Table 2:** Amino acid composition of biomass produced by submerged cultivation of *P. ostreatus* LGAM 1123 on different glucose/xylose mixtures in a 3.5 L stirred tank bioreactor.

	50G0X	35G15X	25G25X	15G35X	0G50X
	mg/g Protein	mg/g Protein	mg/g Protein	mg/g Protein	mg/g Protein
Asp ^1^	49.0 ± 2.3	33.8 ± 10.7	33.7 ± 3.5	23.7 ± 4.4	28.4 ± 11.6
Glu ^2^	42.8 ± 3.1	29.7 ± 4.2	31.1 ± 1.6	29.1 ± 4.5	27.6 ± 3.2
Asn ^3^	3.6 ± 0.8	6.3 ± 0.6	10.4 ± 2.5	0.0	0.0
Gln ^4^	37.7 ± 0.9	43.4 ± 4.9	37.7 ± 2.1	52.9 ± 6.3	53.5 ± 6.4
Ser ^5^	10.2 ± 1.5	15.2 ± 3.8	14.8 ± 1.1	15.9 ± 2.6	20.2 ± 2.9
Gly ^6^	282.2 ± 4.2	264.6 ± 1.2	252.2 ± 6.7	248.2 ± 18.5	246.5 ± 3.5
Val ^7^	82.4 ± 2.6	77.3 ± 3.0	68.9 ± 0.8	71.9 ± 2.3	69.3 ± 0.2
Pro ^8^	143.5 ± 5.1	154.5 ± 30.8	143.8 ± 18.3	143.9 ± 2.8	142.2 ± 9.6
Arg ^9^	59.9 ± 10.2	71.6 ± 0.2	61.6 ± 0.1	78.3 ± 1.1	68.7 ± 2.8
Met ^10^	2.5 ± 0.5	10.9 ± 1.2	12.2 ± 0.6	12.6 ± 2.0	16.1 ± 3.5
Ile ^11^	47.1 ± 2.3	51.1 ± 0.3	53.2 ± 0.4	54.9 ± 2.5	54.0 ± 1.9
Leu ^12^	103.5 ± 3.5	89.1 ± 1.9	97.3 ± 2.9	99.2 ± 3.5	91.7 ± 1.9
Trp ^13^	46.5 ± 1.5	49.7 ± 1.9	45.4 ± 3.0	46.6 ± 0.9	42.8 ± 6.6
Phe ^14^	66.7 ± 0.1	84.2 ± 18.4	69.5 ± 2.3	81.0 ± 6.3	93.7 ± 7.7
Cys ^15^	5.7 ± 0.7	7.8 ± 1.5	4.4 ± 0.1	6.8 ± 2.2	8.6 ± 5.6
Lys ^16^	2.9 ± 0.3	2.8 ± 0.5	1.7 ± 0.6	2.1 ± 0.4	2.9 ± 1.0
Tyr ^17^	3.4 ± 2.1	5.0 ± 1.6	9.5 ± 5.1	6.4 ± 0.1	15.8 ± 0.6
AAA (Phe + Tyr) ^18^	70.1 ± 2.3	89.2 ± 20.0	79.0 ± 7.4	87.4 ± 6.1	109.5 ± 8.3
SAA (Met + Cys) ^19^	8.3 ± 1.1	18.7 ± 0.3	16.5 ± 0.5	19.4 ± 4.2	24.8 ± 9.1
Total	989.9 ± 0.0	989.9 ± 9.3	989.9 ± 9.3	989.9 ± 9.3	989.9 ± 9.3

^1^ aspartic acid, ^2^ glutamic acid, ^3^ asparagine, ^4^ glutamine, ^5^ serine, ^6^ glycine, ^7^ valine, ^8^ proline, ^9^ arginine, ^10^ methionine, ^11^ isoleucine, ^12^ leucine, ^13^ tryptophan, ^14^ phenylalanine, ^15^ cysteine, ^16^ lysine, ^17^ tyrosine, ^18^ AAA: aromatic amino acids, ^19^ SAA: sulfur amino acids.

**Table 3:** Composition of aspen hydrolysate as determined by HPLC analysis.

Aspen Hydrolysate	C (g/L)
Glucose	67.7 ± 0.41
Xylose	41.31 ± 0.18
Mannose	4.18 ± 0.21
Cellobiose	1.89 ± 0.09
Arabinose	1.86 ± 0.09
Galactose	1.77 ± 0.09
Levulinic acid	0.05 ± 0.004
HMF	0.10 ± 0.01
Furfural	0.73 ± 0.17
Acetic acid	6.01 ± 0.005
Formic acid	3.98 ± 0.06
Ethanol	11.63 ± 0.06

## Data Availability

Data is contained within the article or Supplementary Material.

## Supplementary Materials

The following supporting information can be downloaded at: https://www.mdpi.com/article/10.3390/foods12122295/s1. Figure S1: PCA Analysis plots for amino acid composition on different glucose/xylose mixtures.
