# Supplementary material for: Mycoprotein Production by Submerged Fermentation of the Edible Mushroom Pleurotus ostreatus in a Batch Stirred Tank Bioreactor Using Agro-Industrial Hydrolysate
Source: Foods. 2023 Jun 7;12(12):2295. doi: 10.3390/foods12122295 (PMC10297003; doi:10.3390/foods12122295)
Supplement: Supplementary file 1 [file foods-12-02295-s001.zip › foods-2409789-supplementary.pdf]

Supplementary material

Mycoprotein production by submerged fermentation of the edible mushroom Pleurotus ostreatus in a batch stirred tank bioreactor using agro-industrial hydrolysate

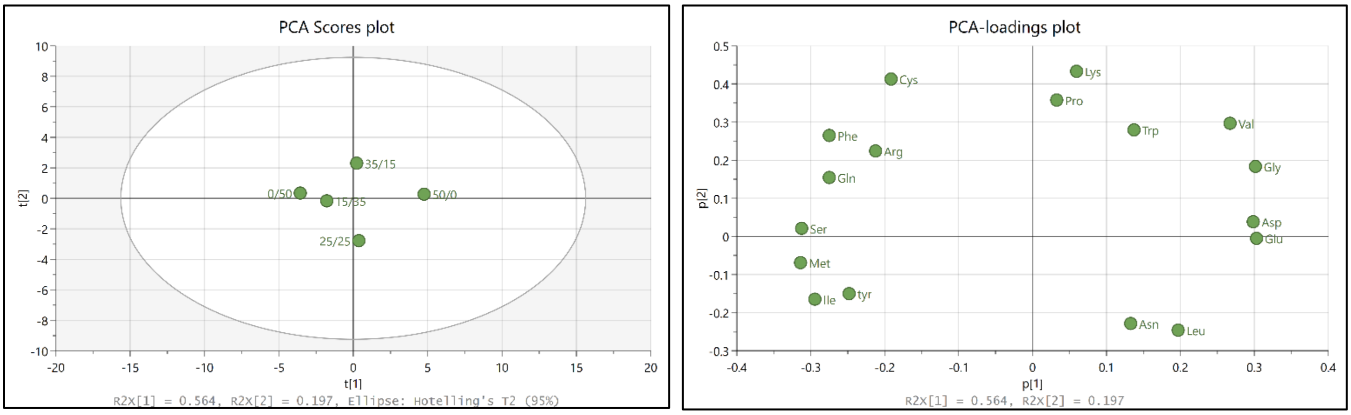

Figure. S1 PCA Analysis plots for amino acid composition on different glucose/xylose mixtures depicting the corresponding loading (on the right) and scores (on the left) plots that establish the relative importance of each variable (amino acids concentrations. 50/0 is 50G0X, 0/50 is 0G50X, 35/15 is 35G15X, 25/25 is 25G25X
